# RRx-001 Radioprotection: Enhancement of Survival and Hematopoietic Recovery in Gamma-Irradiated Mice

**DOI:** 10.3389/fphar.2021.676396

**Published:** 2021-04-22

**Authors:** Kimberly J. Jurgensen, William K. J. Skinner, Bryan Oronsky, Nacer D. Abrouk, Andrew E. Graff, Reid D. Landes, William E. Culp, Thomas A. Summers, Lynnette H. Cary

**Affiliations:** ^1^ Henry M. Jackson Foundation for the Advancement of Military Medicine, Bethesda, MD, United States; ^2^ Scientific Research Department, Armed Forces Radiobiology Research Institute, Uniformed Services University, Bethesda, MD, United States; ^3^ Department of Radiation Oncology, Walter Reed National Military Medical Center, Bethesda, MD, United States; ^4^ EpicentRx, Inc., San Diego, CA, United States; ^5^ Department of Biostatistics, University of Arkansas for Medical Sciences, Little Rock, AR, United States; ^6^ Director, Biomedical Instrumentation Center, Uniformed Services University of the Health Sciences, Bethesda, MD, United States; ^7^ Department of Pathology, Uniformed Services University, Bethesda, MD, United States

**Keywords:** hematopoietic acute radiation syndrome (H-ARS), radiation, countermeasures, RRx-001, total body irradiation (TBI)

## Abstract

The present studies evaluate the *in vivo* prophylactic radioprotective effects of 1-bromoacetyl-3, 3-dinitroazetidine (RRx-001), a phase III anticancer agent that inhibits c-myc and downregulates CD-47, after total body irradiation (TBI), in lethally and sublethally irradiated CD2F1 male mice. A single dose of RRx-001 was administered by intraperitoneal (IP) injection 24 h prior to a lethal or sublethal radiation dose. When irradiated with 9.35 Gy, the dose lethal to 70% of untreated mice at 30 days (LD_70/30_), only 33% of mice receiving RRx-001 (10 mg/kg) 24 h prior to total body irradiation (TBI) died by day 30, compared to 67% in vehicle-treated mice. The same pretreatment dose of RRx-001 resulted in a significant dose reduction factor of 1.07. In sublethally TBI mice, bone marrow cellularity was increased at day 14 in the RRx-001-treated mice compared to irradiated vehicle-treated animals. In addition, significantly higher numbers of lymphocytes, platelets, percent hematocrit and percent reticulocytes were observed on days 7 and/or 14 in RRx-001-treated mice. These experiments provide proof of principle that systemic administration of RRx-001 prior to TBI significantly improves overall survival and bone marrow regeneration.

## Introduction

Ionizing radiation causes damage to normal tissues, ranging from genetic mutations to cell death (Hall and Giaccia, 2012). The harmful effects of ionizing radiation on normal tissues are a major concern for military and emergency responders to nuclear accidents and terrorist events due to the risk of acute and delayed radiation injuries (CDC, 2010). Additionally, radioprotection is a critical issue in cancer treatment. Despite significant technological improvements in radiation delivery in recent years, normal tissue toxicity remains a major dose-limiting factor in therapeutic radiology (Johnke et al., 2014; Nakamura et al., 2014).

Extensive efforts over the past several decades have resulted in two Food and Drug Administration (FDA) approved drugs available for prophylactic radioprotection of non-hematopoietic tissue, amifostine (Ethyol) and palifermin (Kepivance) (Wasserman and Brizel, 2001). However, neither amifostine nor palifermin have been FDA approved for accidental or emergency radiation exposures.

Only three drugs have been FDA approved for the treatment of hematopoietic acute radiation syndrome (H-ARS): 1) Granulocyte colony-stimulating factor (G-CSF, Filgrastim, or Neupogen), 2) Pegfilgrastim (PegG-CSF, Neulasta), and 3) Granulocyte macrophage colony-stimulating factor (GM-CSF, Sargramostim, or Leukine). All work by promoting granulocytes that make up the majority of circulating white blood cells (WBCs) (Mehta et al., 2015). These drugs are radiomitigators and although they have been shown to be effective in multiple animal models of H-ARS, studies have also shown that with prolonged use they may exacerbate radiation induced long-term bone marrow injury, as well as, the long-term recovery from hematopoietic syndrome by promoting hematopoietic stem cell (HSC) proliferation and differentiation leading to HSC exhaustion, in part by promoting HSC senescence (Li et al., 2015).

These limitations require a search for a radioprotective or radiomitigating agent that is deemed sufficiently safe and effective both to shield patients from normal tissue side effects during radiotherapy without simultaneously protecting tumor cells, and to increase survival for the military and first responders in the event of nuclear and radiological emergencies, as well as astronauts exposed to cosmic radiation that would otherwise have been blocked or absorbed by the Earth’s atmosphere.

One minimally toxic option currently under investigation as an anticancer agent in Phase III clinical trials is RRx-001, a small cyclic nitro compound with the IUPAC nomenclature, 1-bromoacetyl-3,3-dinitroazetidine (Ning et al., 2012). Preclinical and clinical research demonstrated that RRx-001 is a vascular normalizer that repolarizes tumor associated macrophages (TAM) from an M2 to M1 phenotype and through c-myc inhibition downregulates expression of the CD47 checkpoint on cancer cells (Cabrales, 2019). These changes effectively mobilize TAMs to seek out and destroy tumor cells, which, in the process, along with improved tumor blood flow, increases susceptibility to chemotherapy and radiation (Ning et al., 2012). RRx-001 is tested clinically to be used as a single agent, and in combination with chemotherapy and/or radiation as a chemo- and radiosensitizer.

Early human phase I and II data have demonstrated broad-spectrum anticancer activity in the absence of typical chemotherapy-related toxicities (Reid et al., 2015). Paradoxically, in non-transformed cells, RRx-001 treatment protects normal tissue from radiation and chemotherapy damage (Ning et al., 2012). *In vivo* studies showed that administration of RRx-001 prior to cisplatin treatment is prophylactic against the development of renal toxicity and chromosomal aberrations (Oronsky et al., 2017). In preclinical testing, RRx-001 (10 mg/kg) given 30 min prior to total body irradiation (10–15 Gy) protected the intestines of C3H mice as shown by an increased number of viable crypt cells (Ning et al., 2012).

The aim of this study was to determine if prophylactic systemic administration of RRx-001 24 h prior to TBI significantly improves overall survival in CD2F1 mice compared to the vehicle control. A 30-day TBI lethality experiment (LD_70/30_ of 9.35 Gy) demonstrated that 24-h prophylactic intraperitoneal (IP) administration of RRx-001 increased overall survival and median survival time in CD2F1 mice. A follow-up experiment to identify the dose reduction factor (DRF) showed that RRx-001 provided a statistically significant DRF of 1.07 compared to the vehicle control. In sublethally TBI mice, prophylactic IP administration of RRx-001 was found to significantly reduce severe reticulocytopenia and leukopenia in addition to improving cellular recovery in bone marrow.

## Materials and Methods

### RRx-001 Drug Preparation

RRx-001 as a powder was obtained from EpicentRx Inc (Mountain View, CA) and formulated according to the manufacturer’s instructions. Briefly, 100% dimethyl sulfoxide (DMSO) (Amresco, Solon, OH) was added to RRx-001, vortexed for 30–60 s and incubated at room temperature for 15 min. Sterile water (Teknova, Hollister, CA) was added to bring the final concentration of DMSO to 5% and the solution vortexed for 2 min. The 2.0 mg/ml RRx-001 stock solution was made up fresh before each experiment. The vehicle control, 5% DMSO in sterile water, was made up following the same procedure. 10 mg/kg RRx-001 or vehicle was injected intraperitoneal (IP) or intravenous (IV) 24 h prior to irradiation or sham-irradiation.

### Mice

Eight-to ten-week-old CD2F1/Hsd male mice were purchased from Envigo (Dublin, VA) and maintained at the Armed Forces Radiobiology Research Institute (AFRRI, Bethesda, MD) vivarium. Mice were allowed to acclimate for at least 7 days prior to initiation of the study. Mice were randomized (3–4/cage) and conventionally housed in sterile polycarbonate boxes with filtered lids (Microisolator, Lab Products Inc., Seaford, DE) and autoclaved hardwood chip bedding. Mice had ad libitum access to Harlan Teklad Rodent diet 8604 (Purina Mills, St. Louis, MO) and acidified water (pH 2.5–3.0). All mice were housed in a controlled environment with a 12-h light-dark cycle, a temperature of 23 ± 2°C, 50 ± 20% relative humidity and 10–15 cycles per hour of fresh air. According to vendor health reports, mice were free of viral, fungal, bacterial, and parasitic adventitial pathogens. All animal procedures were performed in accordance with institutional guidelines, the principles outlined in the National Research Council’s Guide for the Care and Use of Laboratory Animals and approved by AFRRI’s Institutional Animal Care and Use Committee**.**

### Irradiation

Unanesthetized mice were placed in well-ventilated Plexiglas restrainers and irradiated bilaterally at AFRRI’s Cobalt-60 (Co-60) gamma irradiation facility. Sham-irradiated mice were also placed in identical Plexiglas restrainers and kept in a room shielded from irradiation at the same time. In each experiment, the dose to the abdominal cores of the animals was delivered at a dose rate of approximately 0.6 Gy/min. Dosimetry was performed prior to the irradiation of the animals using the highly accurate alanine/electron spin resonance (ESR) dosimetry system (American Society for Testing and Materials, Standard E 1607) to measure dose rates (to water) in the cores of acrylic mouse phantoms, which were located in the compartments of the exposure rack. A calibration curve based on standard alanine calibration dosimeters provided by the National Institute of Standards and Technology (NIST, Gaithersburg, MD) was used to measure the doses. The accuracy of AFRRI’s dose rate calibrations has been verified several times using the services of the National Physics Laboratory (United Kingdom National Standards Laboratory, London, United Kingdom) and the M.D. Anderson Cancer Center (Houston, TX). The corrections applied to the measured dose rates in the phantoms were for a small difference in the Co-60 energy between the mass energy-absorption coefficients for soft tissue and water, as well as source decay. The radiation field was uniform within ±1.2%.

### Radioprotection Survival Study

For the survival study, mice underwent TBI at 9.35 Gy [the LD70/30 (Kumar et al., 2018)] at a dose rate of 0.6 Gy/min using gamma photons. Twenty-four hours prior to irradiation, 12 mice received 10 mg/kg RRx-001; the remaining 12 mice received the vehicle control (5% DMSO in sterile H2O) by IP or IV injection. Mice were monitored at least twice a day for 30 days post-irradiation. During the critical period (days 10–20), mice were monitored at least three times a day with no more than 10 h between observations. Mice displaying any signs of discomfort received food in their cage as a wet mash. Mice displaying overt dyspnea, weight loss, lethargy, or other markers of moribundity and appearing to be in distress were humanely euthanized in a separate room using carbon dioxide gas followed by cervical dislocation after breathing stopped as a confirmatory method of euthanasia. The IV survival study had 24 mice total. The IP survival study was repeated with an additional 24 mice; thus, for final analysis, each of the two treatment groups had 24 mice (48 mice in total).

### Prophylactic Dose Reduction Factor (DRF) Study

We sought to estimate the DRF of RRx-001 prophylactically administered 24 h prior to TBI. Half of the allotted animals were randomized to receive 10 mg/kg of RRx-001, and the other half to receive 5% DMSO in sterile H_2_0 by IP injection. Within each treatment group, we further equally randomized mice among six radiation doses: 7.5, 8, 8.5, 9, 9.5, and 10 Gy for vehicle-treated mice, and 8.5, 9, 9.5, 10, 10.5, and 11 Gy for drug-treated mice. In order to detect a DRF of 1.10 with 0.90 power on a one-sided 0.05 significance level test, we calculated 84 mice (split equally among all 12 treatment-radiation groups) were needed. This calculation assumed an LD50/30 for vehicle mice of 8.9 Gy, the aforementioned radiation doses, and a lethality slope of 25 on log10 radiation dose in a probit regression (Kodell et al., 2010). However, as part of an ongoing feasibility study, we used 16 mice per treatment-radiation group (192 in total), which would allow detection of a DRF = 1.06 with 0.90 power. Mice were monitored at least twice a day post-irradiation, and at least 3 times a day during the critical period (days 7–20) with no more than 10 h between observations. Mice displaying any signs of discomfort received food in their cage as a wet mash. Mice displaying markers of moribundity and appearing to be in distress were humanely euthanized as described previously. Survival to 45 days after irradiation was recorded and survival to 30 days was analyzed.

### Hematopoietic Study

To determine the pathophysiological effects of RRx-001 on hematopoietic protection in mice, a sublethal dose of TBI (7 Gy at 0.6 Gy/min using Co-60) was used (Ghosh et al., 2009). Sixty (60) CD2F1 male mice were randomized into four experimental groups: 1) irradiation + vehicle, 2) irradiation + RRx-001, 3) sham-irradiation + vehicle, and 4) sham-irradiation + RRx-001. Either 10 mg/kg RRx-001 or the vehicle control were IP injected 24 h prior to either irradiation or sham-irradiation (day 0). The 15 mice within each group were then randomized (3 mice/group/timepoint) to be humanely euthanized on days 2, 7, 14, 21, and 28 post-irradiation (day 0). Blood was removed by cardiocentesis using a 1 ml syringe with a 23 g needle in mice anesthetized with 3–5% isoflurane (Baxter, Deerfield, IL). This was performed as a terminal procedure, and followed by cervical dislocation as a confirmatory method of euthanasia. A portion of the sample was immediately transferred into EDTA tubes (Sarstedt Inc., Newton, NC) and gently rotated until the time of analysis. The tubes were analyzed for a complete blood count with differential and reticulocytes using the ADVIA 2120 (Siemens Medical Solutions Diagnostics, Dublin, Ireland), and Microsoft software version 5.9 (Microsoft Corp., Redmond, WA) to generate the data. Serum was separated from the rest of the blood sample for an enzyme-linked immunosorbent assay (ELISA), and bone marrow and sternebrae were then collected. These procedures were repeated with an additional 60 mice; thus, providing *n* = 6 mice/group/time point (120 mice in total) for analysis.

### Sternum Marrow Histopathology

Sternebrae collected during the hematopoietic study were fixed in 10% zinc-buffered formalin for at least 24 h and up to 7 days. Fixed sternebrae were decalcified for 3 h in 12–18% sodium EDTA (pH 7.4–7.5) and specimens dehydrated using graded ethanol concentrations and embedded in paraffin. Longitudinal 4 μm sections were stained with regular hematoxylin and eosin. Two board-certified pathologists conducted histopathological evaluation of the samples. One of the pathologists scored all the samples blindly. Bone marrow was evaluated *in situ* within sternebrae and graded (grade 1: ≤10%; grade 2: 11–30%; grade 3: 31–60%; grade 4: 61–89%; grade 5: ≥90%) for total cellularity. Megakaryocytes were also quantified based on the average per 10 high power fields (HPF) at 400× magnification using a BX43 or BX53 microscope (Olympus, Minneapolis, MN). Images were captured with an Olympus DP22 camera and imported into Olympus cellSens Standard software for review.

### Colony Forming Unit Assay

Bone marrow was collected from both femurs of CD2F1 mice during the hematopoietic study as described previously (Kumar et al., 2018) and were pooled so that *n* = 3 mice/group/day. The pooled bone marrow cells were suspended in semisolid cultures using 1 ml of MethoCult™ GF+ system for mouse cells per 35-mm cell culture dish and plated in triplicate (Stem Cell Technologies, Vancouver, BC, Canada) according to the manufacturer’s instructions. Sham-irradiated groups were plated at 1 × 10^4^ cells. Irradiated (7 Gy) groups were plated at 4 × 10^4^ cells. All colonies were counted 12 days after incubation and 50+ cells constituted one colony. Data is represented as the mean ± SEM of *n* = 3 mice/group/time point for a total of 57 animals.

### Circulating Levels of G-CSF

Serum collected from the hematopoietic study was used to detect circulating levels of G-CSF using the mouse G-CSF Quantikine ELISA kit (R&D Systems, Minneapolis, MN) and following manufacturer’s instructions. The cytokine detection limit was 5 pg/ml and the G-CSF positive control was between 97.9–163.2 pg/ml. Data is represented as the mean ± SEM of *n* = 2–3 mice/group/time point for a total of 50 animals.

### Statistical Analyses

For the survival study, estimated survival curves were produced with Kaplan-Meier’s method, and compared with a two-sided log-rank test. For the DRF study, we estimated the DRF and its 95% confidence interval with probit regression of mouse mortality on treatment and log10-transformed radiation dose as described elsewhere (Landes et al., 2013). For the pathophysiology study, blood and bone marrow parameters were estimated and compared between treatment groups using analysis of variance (ANOVA), with treatment and euthanasia day as factors. In the ANOVA of bone marrow parameters, pathology was included as a factor. Finally, to determine whether ANOVA results depended on normal assumptions, we also compared treatment groups using nonparametric analogues of the ANOVAs, such as Wilcoxon–Mann–Whitney tests and Kruskal–Wallis analyses as sensitivity analyses.

For all analyses, *p* < 0.05 was considered significant; we also present 95% confidence intervals. Given the nature of the experiments, sample size determination based on the power to detect group differences was not an a-priori consideration, the multiplicity of comparisons impact on significance is deemed unimportant since most *p* values were highly significant. We used R software (Version 3.4.3, 2016) and SAS/STAT software, version 9.4, SAS System for Windows (SAS Institute, Cary, NC, United States) for analyses, and R software and GraphPad Prism version 7.03 (GraphPad Software, La Jolla, CA) for figures. Data and software code for producing the results in this work are available upon request.

## Results

### Pretreatment With RRx-001 Increases Survival After Lethal Total Body Irradiation

**FIGURE 1:**
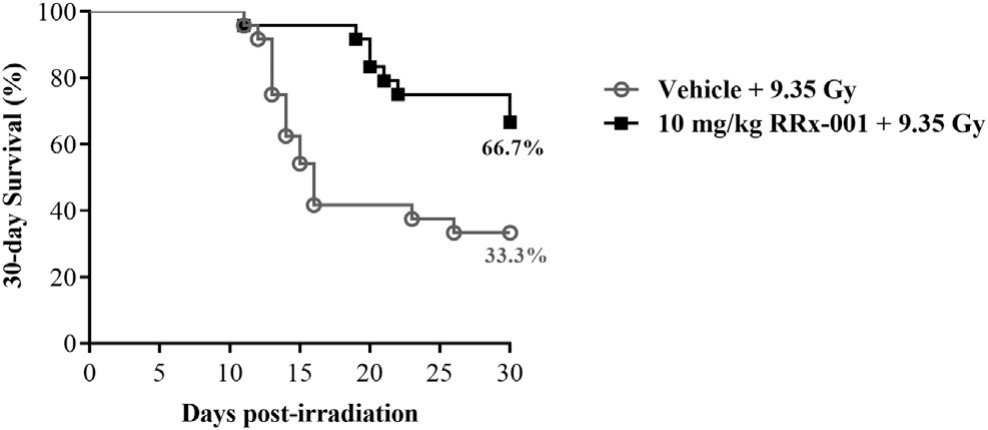
Kaplan–Meier 30-day survival curves illustrating the increased and prolonged survival of CD2F1 mice prophylactically treated by IP injection with 10 mg/kg RRx-001 compared to the vehicle control, 24 h prior to 9.35 Gy whole body irradiation; log-rank $\chi_{\left(1\right)}^{2}$ = 7.65, *p* = 0.006. *N* = 12 mice/group (24 mice total) per experiment. The experiment was performed in duplicate (48 mice total).

Survival improvement in favor of IP pretreatment with one dose of 10 mg/kg RRx-001 over vehicle control in mice 24 h prior to a lethal TBI of 9.35 Gy was highly significant: 67% of RRx-001 treated animals survived to 30 days compared to 33% of vehicle treated animals (log-rank $\chi_{\left(1\right)}^{2}$ = 7.65, *p* = 0.006; Figure 1). The results also demonstrated that 24-h prophylactic IP administration of RRx-001 increased median survival time by at least 14 days over that for vehicle treated animals. When mice were administered 10 mg/kg RRx-001 or vehicle by IV injection 24 h prior to a lethal TBI of 9.35 Gy, survival was significantly higher after 30 days in the RRx-001 treated mice: 50% survival in RRx-001 treated mice vs. 8% survival in vehicle treated mice (log-rank $\chi_{\left(1\right)}^{2}$ = 5.62, *p* = 0.018; Supplementary Figure S1).

### Determination of DRF Showed RRx-001 Increases Resistance to Radiation Lethality

**FIGURE 2:**
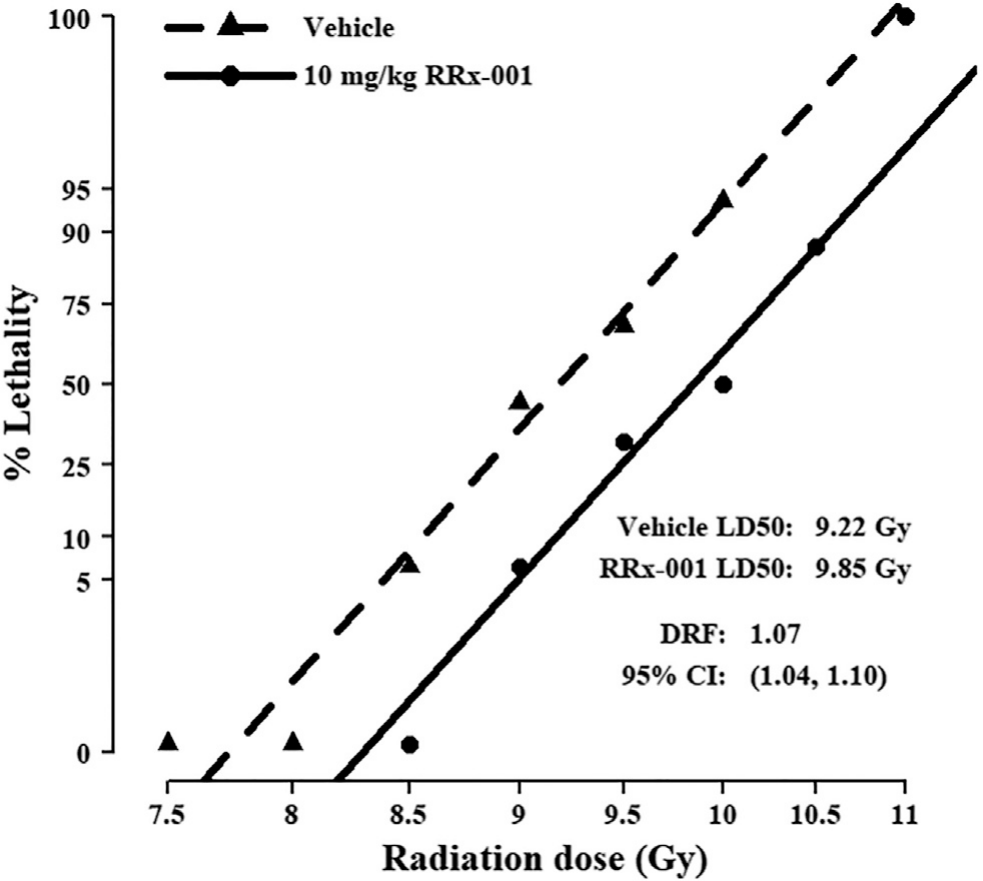
Probit mortality curves for 192 mice equally randomized to prophylactic treatment with 10 mg/kg RRx-001 or vehicle control; mice were further equally randomized among the six indicated TBI doses to determine the DRF (*n* = 16 mice/group). LD_50/30_ and DRF were estimated with probit regression of mortality on log_10_ dose of radiation. The LD_50/30_ for vehicle pre-treated mice was 9.22 (95% CI: 9.03, 9.41), and for RRx-001 pre-treated mice was 9.85 (95% CI: 9.66, 10.05); thus, the DRF was 1.07 (95% CI: 1.04, 1.10). The common slope was 39.6 (95% CI: 29.8, 49.4).

Before estimating the DRF, we first checked the assumption of a common slope on radiation dose for the vehicle- and RRx-001-treated groups from the initial survival study results, each having a slope of 37.6 and 41.5, respectively. This difference of 3.8 in slopes is negligible based on the 95% confidence intervals (CI: −15.9, 23.5). Fitting the common-slope model, the LD_50/30_ for mice treated prophylactically with RRx-001 was 9.85 Gy, and for vehicle-treated mice was 9.18; thus, the DRF was 1.07 (CI: 1.04, 1.10) (Figure 2). In the IP survival study, 2 RRx-001-treated mice died on day 30; therefore, mice were monitored for survival out to day 45. On day 34, one vehicle-treated mouse in the 10 Gy group was the only casualty between day’s 31–45.

### RRx-001 Reduced Pancytopenia After Sublethal Irradiation

In both pretreatment groups of mice, an acute irradiation at a sublethal dose induced severe reticulocytopenia and leukopenia (Table 1). Reticulocyte levels reached a nadir around day 7, increased to levels notably above baseline at day 21, then returned to near-baseline levels by day 28. Throughout the fluctuation over the first 21 days, RRx-001 pretreated mice were estimated to be closer to baseline levels than those pretreated with vehicle. White blood cells and lymphocytes also reached their nadir around day 7, and though levels started to increase, they did not reach baseline levels by day 28. However, white blood cells and lymphocyte levels in irradiated mice pretreated with RRx-001 tended to increase faster than that of their vehicle-pretreated peers. After irradiation, levels of neutrophils and platelets reached their nadir by day 7, and remained at low cellular levels through day 14. From days 7–14, levels in the RRx-001 pretreated mice did not drop as low as that in the vehicle-pretreated mice. After day 14, neutrophil and platelet levels in both groups improved over the next two weeks. Percent hematocrit reached its lowest point 14 days after irradiation, with the level in RRx-001 pretreated mice remaining slightly higher than that in vehicle-pretreated mice. Hematocrit levels were back to baseline levels by day 21 in both pretreatment groups.

**TABLE 1:** Prophylactic treatment with RRx-001 Reduced Pancytopenia after Sublethal Irradiation.

Time after radiation	Treatment group	WBC (×10^3^ cells/µL)	ALC (×10^3^ cells/µL)	ANC (×10^3^ cells/µL)	Platelets (×10^3^ cells/µL)	% Hematocrit	% Reticulocytes
Average days 2, 7, 14, 21, 28	Vehicle + sham	5.01 ± 0.31	3.75 ± 0.26	0.91 ± 0.06	913.66 ± 52.55	41.59 ± 0.44	2.54 ± 0.07
	RRx-001 + Sham	4.73 ± 0.27	3.38 ± 0.21	0.97 ± 0.06	**1,067.73 ± 32.26	41.66 ± 0.45	2.62 ± 0.07
Day 2	Vehicle +7 Gy	0.82 ± 0.14	0.07 ± 0.01	0.67 ± 0.13	863.00 ± 88.08	39.27 ± 0.85	ND
	RRx-001 + 7 Gy	1.38 ± 0.20	0.07 ± 0.01	1.13 ± 0.20	979.00 ± 40.35	41.07 ± 0.82	ND
Day 7	Vehicle +7 Gy	0.18 ± 0.05	0.08 ± 0.03	0.07 ± 0.01	93.33 ± 16.29	30.08 ± 0.99	0.03 ± 0.01
	RRx-001 + 7 Gy	0.21 ± 0.03	0.04 ± 0.00	*0.11 ± 0.02	*133.40 ± 16.74	31.36 ± 1.00	0.05 ± 0.01
Day 14	Vehicle +7 Gy	0.25 ± 0.03	0.18 ± 0.02	0.06 ± 0.01	78.33 ± 9.97	25.27 ± 0.62	1.62 ± 0.28
	RRx-001 + 7 Gy	*0.70 ± 0.20	*0.49 ± 0.14	*0.14 ± 0.03	*143.25 ± 36.54	*28.38 ± 0.55	*4.05 ± 1.08
Day 21	Vehicle +7 Gy	1.19 ± 0.13	0.51 ± 0.08	0.52 ± 0.04	371.60 ± 66.29	40.40 ± 0.36	7.08 ± 1.59
	RRx-001 + 7 Gy	1.11 ± 0.11	0.46 ± 0.03	0.50 ± 0.07	448.33 ± 40.37	39.38 ± 1.58	6.06 ± 1.03
Day 28	Vehicle +7 Gy	2.20 ± 0.34	0.77 ± 0.19	1.17 ± 0.20	595.50 ± 135.15	35.83 ± 2.18	2.62 ± 0.09
	RRx-001 + 7 Gy	2.69 ± 0.33	1.18 ± 0.32	1.09 ± 0.11	497.60 ± 116.12	39.02 ± 1.57	*3.66 ± 0.40

Values are the mean ± SEM (*n* = 2–3 mice/group/time point); ND, not determined. *p < 0.05 comparing the irradiated groups/time point. Significance was determined using a parametric test consisting of a general linear model ANOVA and the Kruskal–Wallis nonparametric test. **p < 0.01 comparing the nonirradiated groups combining days 2, 7, 14, 21 and 28. Significance was determined using the longitudinal mixed model repeated measures analysis. This experiment was performed in duplicate.

### RRx-001 Increases Bone Marrow Recovery After Irradiation

To determine the effect of RRx-001 on bone marrow, a histopathological analysis of bone marrow sternebrae was performed and the cellularity, as reported by grade (grade 1: ≤10%; grade 2: 11–30%; grade 3: 31–60%; grade 4: 61–89%; grade 5: ≥90% cellularity), and megakaryocyte numbers (averaged per 10 high-powered fields; HPF) were ascertained by two pathologists, one of which scored all the samples blindly (TAS, WEC). In determining significance for grade of cellularity and average number of megakaryocytes per 10 HPF, the differences between pathologists and the interaction between treatment and pathologist were not significantly different.

**FIGURE 3:**
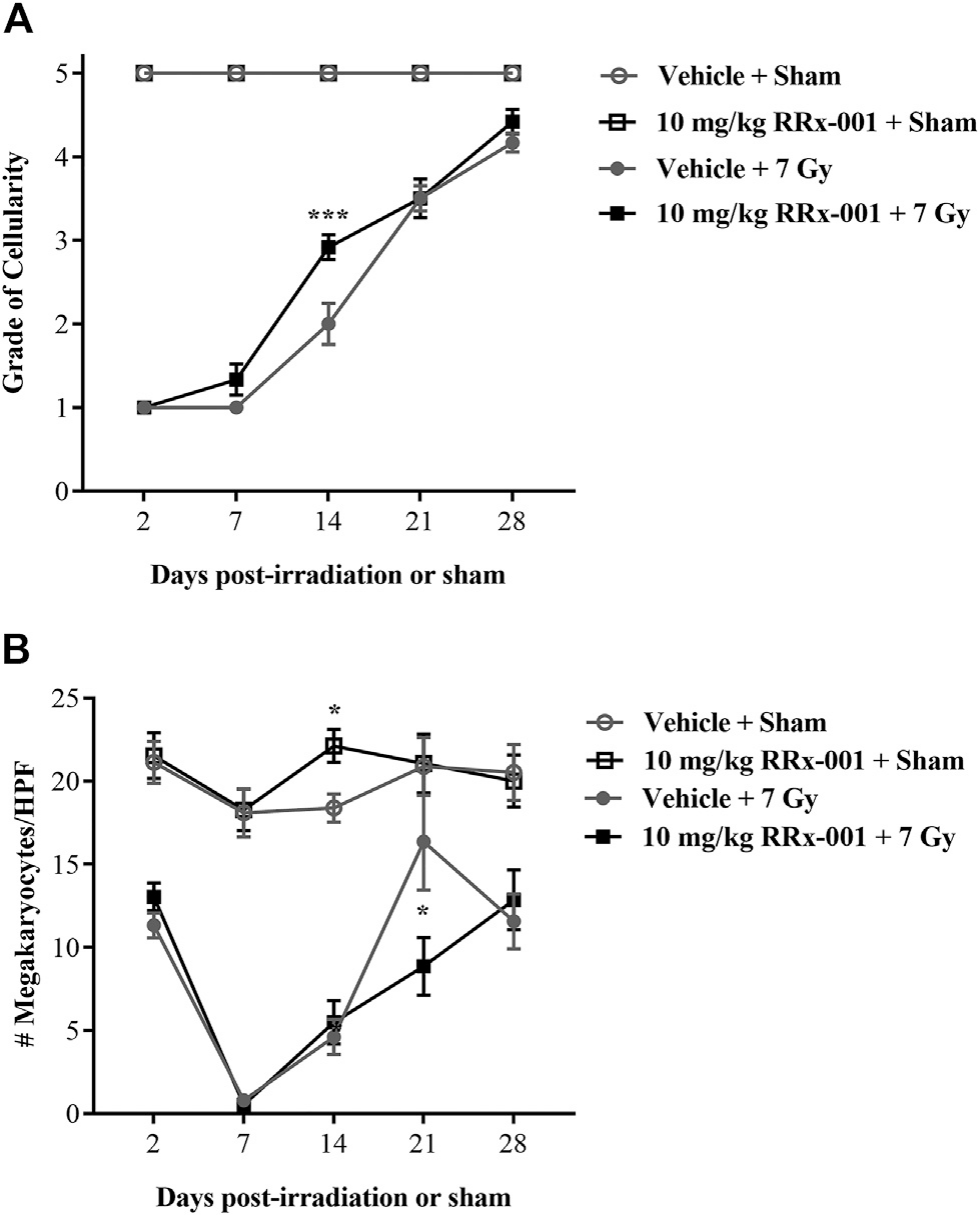
RRx-001 significantly increased the grade of bone marrow cellularity but not megakaryocytes after sublethal total body irradiation **(A)** Pretreatment with 10 mg/kg RRx-001 significantly increased the grade of bone marrow cellularity (grade 1: ≤10%; grade 2: 11–30%; grade 3: 31–60%; grade 4: 61–89%; grade 5: ≥90%) on day 14 after irradiation compared to the vehicle control; ***p < 0.0001 **(B)** In the sham-treated groups, RRx-001 had a significant increase in average megakaryocyte number per 10 HPF compared to the vehicle control on day 14; *p = 0.041. However, in the irradiated groups on day 21 there was a significant spike in megakaryocyte numbers in the vehicle-treated group compared to the RRx-001-treated group; *p = 0.029. Error bars show mean ± SEM. Significance was determined using a parametric test consisting of a general linear model ANOVA and the Kruskal–Wallis nonparametric test. *N* = 3 mice/group/time point (60 mice total) per experiment. The experiment was performed in duplicate (120 mice total).

The overall cellularity of the bone marrow in both the sham-irradiated RRx-001- and vehicle-pretreated groups never dropped below 90% during the duration of the study and therefore maintained a grade of 5 (Figure 3A). As expected after irradiation, both the RRx-001- and vehicle-pretreated groups had a massive loss in bone marrow cellularity, with all mice having grade 1 cellularity by day 2 after radiation. At day 7, a slight increase in cellularity was observed by the pathologists in the irradiated, RRx-001-pretreated mice compared to the irradiated, vehicle-pretreated mice. Irradiated mice pretreated with RRx-001, significantly accelerated hematopoietic recovery as determined by the grade of bone marrow cellularity compared with vehicle-pretreated, irradiated mice on day 14. By day 21, both vehicle- and RRx-001-treated irradiated mice displayed equivalent bone marrow cellularity, and improvement continued in both pretreatment groups through day 28; however, neither irradiated group reached cellularity grade 5.

Counts of megakaryocytes in sham-irradiated mice were similar over 28 days for the two pretreatment groups, except at day 14 when RRx-001 pretreated mice had more than vehicle-pretreated mice (Figure 3B). Irradiation-induced decreases in megakaryocyte counts were evident at day 2 in the two pretreatment groups, and continued to drop to near 0 by day 7. However, after day 7, both pretreatment groups of irradiated mice started recovering toward baseline levels of megakaryocytes through day 28, but failed to fully recover to the levels of sham-irradiated mice. Interestingly, the vehicle-pretreated mice had more megakaryocytes than RRx-001 pretreated mice at day 21, but these two groups were similar again by day 28.

**FIGURE 4:**
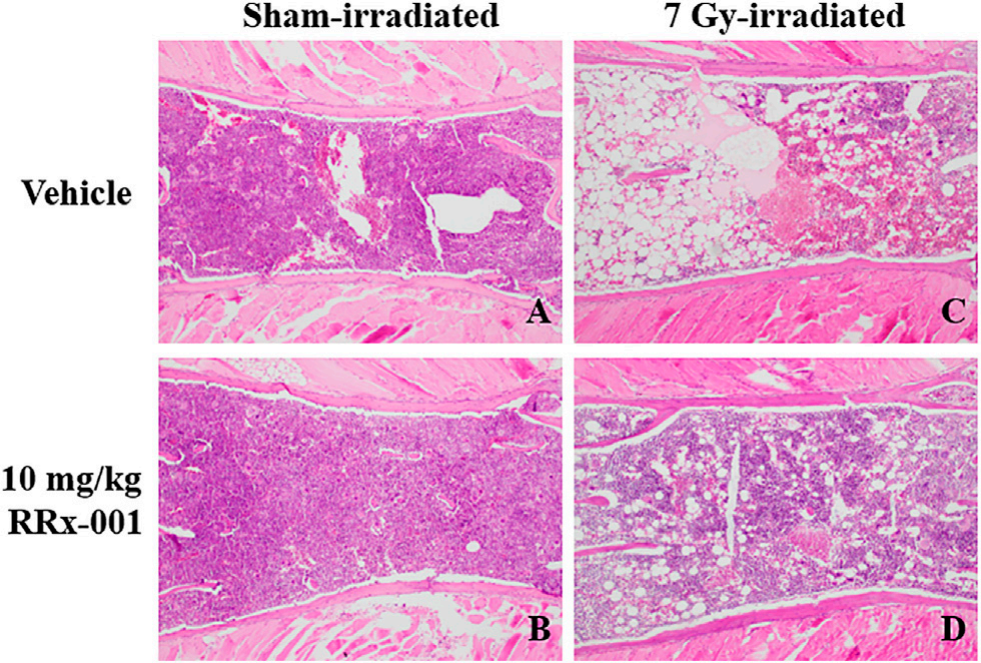
Photomicrographs of H&E stained thin sections of bone marrow sterna showing representative bone marrow cellularity in all groups on day 14. Panels **(A)** and **(B)**: Bone marrow sterna pretreated with either vehicle or 10 mg/kg RRx-001 on day 14 after sham-irradiation. Panel **(C)**: Vehicle-treated sternal bone marrow 14 days after sublethal irradiation illustrated severe hypocellularity as evident by marrow space demonstrating only residual stromal fat (visualized as increased marrow white space). Panel **(D)**: RRx-001-treated sternal bone marrow 14 days after sublethal irradiation showed significantly increased cellularity as seen by the increased amounts of cells stained purple and decrease in the visualized stromal adipose tissue (white space) (×100 magnification).

Figure 4 depicts representative bone marrow histopathology from each experimental group at day 14, where 4A and 4B denote normal bone marrow morphology and cellularity in sham-irradiated mice, treated with either vehicle or RRx-001, respectively. The irradiated vehicle-treated group (4C) showed a significant loss of bone marrow cellularity with an increase in infiltration by adipocytes compared to the irradiated RRx-001-treated group (4D) on day 14 where significant recovery of bone marrow cellularity was observed.

## Discussion

This is the first study to report that RRx-001 administered IP 24 h prior to an LD_70/30_ dose of TBI significantly decreased and delayed lethality in CD2F1 mice. In this study, 33% of mice receiving vehicle before undergoing irradiation with the LD_70/30_ dose survived to 30 days, compared to 67% of the mice pretreated with RRx-001 (Figure 1). RRx-001 extended the median survival time by at least 14 days over the median survival time for vehicle-treated mice. We chose to use the IP method of drug administration to maintain consistency with the previous findings, however similar results were seen when mice were administered RRx-001 by IV injection 24 h prior to radiation (Supplementary Figure S1). This demonstrates that RRx-001 efficacy is not dependent on route of administration. Moreover, in the sublethally irradiated CD2F1 mice, RRx-001 reduced radiation augmented cellular recovery in bone marrow.

The hallmark of the acute radiation syndrome and hematopoietic subsyndrome is pancytopenia and bone marrow failure. A sharp decrease, or complete depletion of bone marrow cells is likely to cause death due to severe immunosuppression (Hall and Giaccia, 2012). To determine the underlying RRx-001-mediated mechanism of protection against H-ARS, mice were sublethally irradiated and humanely sacrificed at various time points to collect blood and bone marrow. In both the vehicle- and RRx-001-treated irradiated groups, myelosuppression was observed, along with severe reticulocytopenia and leukopenia up until day 14. However, significantly higher levels of leukocytes, reticulocytes and platelets on day 14 were seen in the irradiated RRx-001-treated group as compared to the irradiated vehicle control. In addition, the level of neutrophils in RRx-001 treated mice were significantly higher on both days 7 and 14 compared to the vehicle control (Table 1). Increased bone marrow cellularity was observed on day 14 post-irradiation in the RRx-001 treatment group compared to the vehicle control (Figures 3, 4). In addition, improvement in bone marrow hematopoietic progenitor cell recovery was observed in RRx-001 treated irradiated mice, compared to vehicle treated irradiated mice by day 21 (Supplementary Figure S2). Collectively, these results suggest that the cellular protection mechanism of RRx-001 involves accelerated hematopoietic regeneration or mobilization of leukocytes, reticulocytes and platelets, which prevented or delayed mortality from infection and sepsis. Alternatively, and/or in addition to accelerated myeloreconstitution, RRx-001 may have protected hematopoietic stem and progenitor cells.

In previous studies, RRx-001 has shown the ability to increase reactive oxygen and nitrogen species (RONS) (Ning et al., 2012) and activate compensatory responses that enable the cells and tissues to better withstand the deleterious effects of subsequent exposure to higher levels of RONS (Cabrales et al., 2017). The major difference between RRx-001 and other potential redox-active radioprotectors is that RRx-001 has demonstrated minimal toxicity and systemic deliverability (Reid et al., 2015) as well as evidence of anti-cancer activity and potential radiochemoprotection in multiple tumor types such as small-cell lung carcinoma and melanoma brain metastases (Carter et al., 2016) (Kim et al., 2016). Pretreatment paradigms with low-level oxidative stressors have been previously and extensively described in cell-survival studies from yeast, mammalian and human cells *in vitro*, as well as in animal models *in vivo* (Wolff et al., 1988). RRx-001 appears to increase DNA repair activity through activation of the nuclear factor erythroid 2-related factor (Nrf2) transcription factor, which regulates antioxidant response element (ARE) genes (Ning et al., 2015). RRx-001 may also modulate the activation of p53 (Das et al., 2016), thereby altering the response of cells to DNA damage and potentially reducing apoptosis and/or senescence. The increase in ARE via Nrf2 activation (Ning et al., 2015) provides an intriguing mechanism of action for radioprotection. In support of this, preliminary *in vitro* studies from our lab provided evidence RRx-001 treatment increased heme oxygenase-1 (HO-1), an Nrf2 activated antioxidant response element, protein expression in mesenchymal stem cells, monocytes, and macrophages (data not shown). Work by Hedblom et al., confirmed that HO-1 functions to prevent DNA damage, reduce senescence, improve proliferation, and modulate cytokine expression in macrophages (Hedblom et al., 2019). Additional reports, including one from an HO-1 deficient patient, highlight the role of HO-1 in cellular protection against inflammation and oxidative stress (Yachie et al., 1999).

Recombinant G-CSF is utilized in the clinic to attenuate chemotherapeutic-induced toxicity, and is one of three FDA approved medical countermeasures for ARS. However, in contrast to regulated administration of G-CSF, low level endogenous G-CSF expression is typical in healthy individuals and mice, and G-CSF elevation in serum is correlated with inflammation, infection, and tissue damage, and accompanies additional inflammatory stimuli such as interleukin (IL)–1β, and tumor necrosis factor-alpha. G-CSF levels subsequently decline with recovery [reviewed in (Theron et al., 2020)]. Increased circulating G-CSF in response to radiation exposure was previously demonstrated and was not dose rate dependent (Kiang et al., 2018), or radiation quality dependent (Ossetrova et al., 2018). We saw modulation of serum G-CSF in irradiated RRx-001 treated mice (Supplementary Figure S3). Based on our data, RRx-001 may reduce inflammation and tissue damage. Further studies testing the circulating levels of IL-1β, IL-6 or other pro-inflammatory cytokines would support our finding. Taken together, it appears that RRx-001 treatment reduced inflammation, accelerated bone marrow recovery, and aided in immune health recovery after radiation. Most of the effects thought to promote survival occurred at or beyond day 14. Further kinetic studies would be important to optimize dosing of RRx-001 for radioprotection. Careful scientific design might allow the investigation of the relevance of RRx-001 mediated upregulation of HO-1 expression in radioprotection in our model.

Amifostine was initially developed by the United States Army as part of their nuclear warfare program (Wasserman and Brizel, 2001; Koukourakis, 2002). Animal experiments demonstrated radioprotective effects to the skin, mucosa, hair follicles, intestinal wall, and salivary glands (Koukourakis, 2002) and subsequently lead to its use in randomized control trials. Several trials demonstrated that amifostine reduced acute and chronic xerostomia in head and neck cancer patients treated with chemoradiation (Brizel et al., 2000; Veerasarn et al., 2006; Gu et al., 2014). However, due to amifostine’s unacceptable adverse effect profile at therapeutic doses, it is not often used clinically. The second drug, palifermin, is a modified version of keratinocyte growth factor (KGF) (Wasserman and Brizel, 2001; Johnke et al., 2014) that is approved to reduce oral mucositis. In 2015, G-CSF was the first FDA approved countermeasure for the management of H-ARS under the FDA Animal Rule. This rule is intended to facilitate the development of new drugs and biologic products as medical countermeasures for chemical, biological, radiological, and nuclear threats when human efficacy studies cannot be ethically conducted (Food and Drug Administration, 2002; Farese and MacVittie, 2015). G-CSF increased production, effector differentiation, and early release of neutrophils, thereby reducing the duration of severe neutropenia, and minimizing the risk of bacteremia and sepsis (Mehta et al., 2015). G-CSF has been shown to increase survival in non-human primates who were exposed to lethal (myelosuppressive) doses of radiation within the H-ARS model (Farese et al., 2013; Farese et al., 2014). PegG-CSF is a modified, pegylated form of G-CSF with an increased plasma half-life, allowing for less frequent dosing than G-CSF. PegG-CSF has also been shown to improve survival in non-human primates after exposure to lethal doses of radiation (Hankey et al., 2015). The most recent FDA approved countermeasure for H-ARS is GM-CSF (Singh and Seed, 2018).

Even though there has been an overwhelming amount of data available to substantiate the efficacy of these FDA approved radioprotectors and radiomitigators, each has their own limitations and scope of use. The major limitation of amifostine is its serious adverse side effect profile, which results in high rates of discontinuation when given in conjunction with head and neck radiotherapy (Rades et al., 2004). In contrast, palifermin, has been shown to have a well-tolerated toxicity profile; however, its major limitation is its narrow scope of use, which is restricted to the improvement of oral mucositis as demonstrated by a randomized control trial in patients who received total body irradiation as part of a conditioning regimen prior to stem cell transplant (Spielberger et al., 2004). In addition, palifermin is also administered intravenously for three consecutive days making it an unlikely candidate for emergency use. Although G-CSF, pegG-CSF and GM-CSF have been shown to be effective in multiple animal models of H-ARS, they are radiomitigators and would therefore not provide protection for military and first responders in the event of nuclear and radiological emergencies, as well as astronauts before incoming bursts of increased cosmic radiation. In addition, studies have shown that prolonged use can affect long-term recovery from hematopoietic syndrome by promoting hematopoietic stem cell (HSC) proliferation and differentiation leading to HSC exhaustion, partly from promoting HSC senescence and may also may exacerbate radiation induced long-term bone marrow injury (Li et al., 2015).

In conclusion, the increasing potential of terrorists with dirty bombs, nuclear power plant accidents, rogue states with nuclear weapons and long-range delivery systems, as well as the exciting possibility of deep space travel, all continue to present a significant risk of dangerous radiation exposure. Therefore, effective radioprotection is a clear unmet need that RRx-001, as a possible dual-purpose agent with applications in clinical oncology, radiation accidents, nuclear weapons incidents, terrorism, space travel, and radiation site cleanup, has the potential to address. As this study is a proof-of-concept, it is limited by the selection of one 10 mg/kg dose of RRx-001, one time point of 24 h prior to TBI, and IP injection, which would not be the intended human route of administration. Dose, time and route of administration optimization studies in the small animal model, as well as future studies in additional animal models, combined with mechanistic studies in appropriate *in vitro* models are required to support RRx-001 as a medical countermeasure candidate.

## Data Availability

The raw data supporting the conclusion of this article will be made available by the authors, without undue reservation.
