# Supplementary figures and images for: RRx-001 Radioprotection: Enhancement of Survival and Hematopoietic Recovery in Gamma-Irradiated Mice
Source: Front Pharmacol. 2021 Apr 22;12:676396. doi: 10.3389/fphar.2021.676396 (PMC8100686; doi:10.3389/fphar.2021.676396)

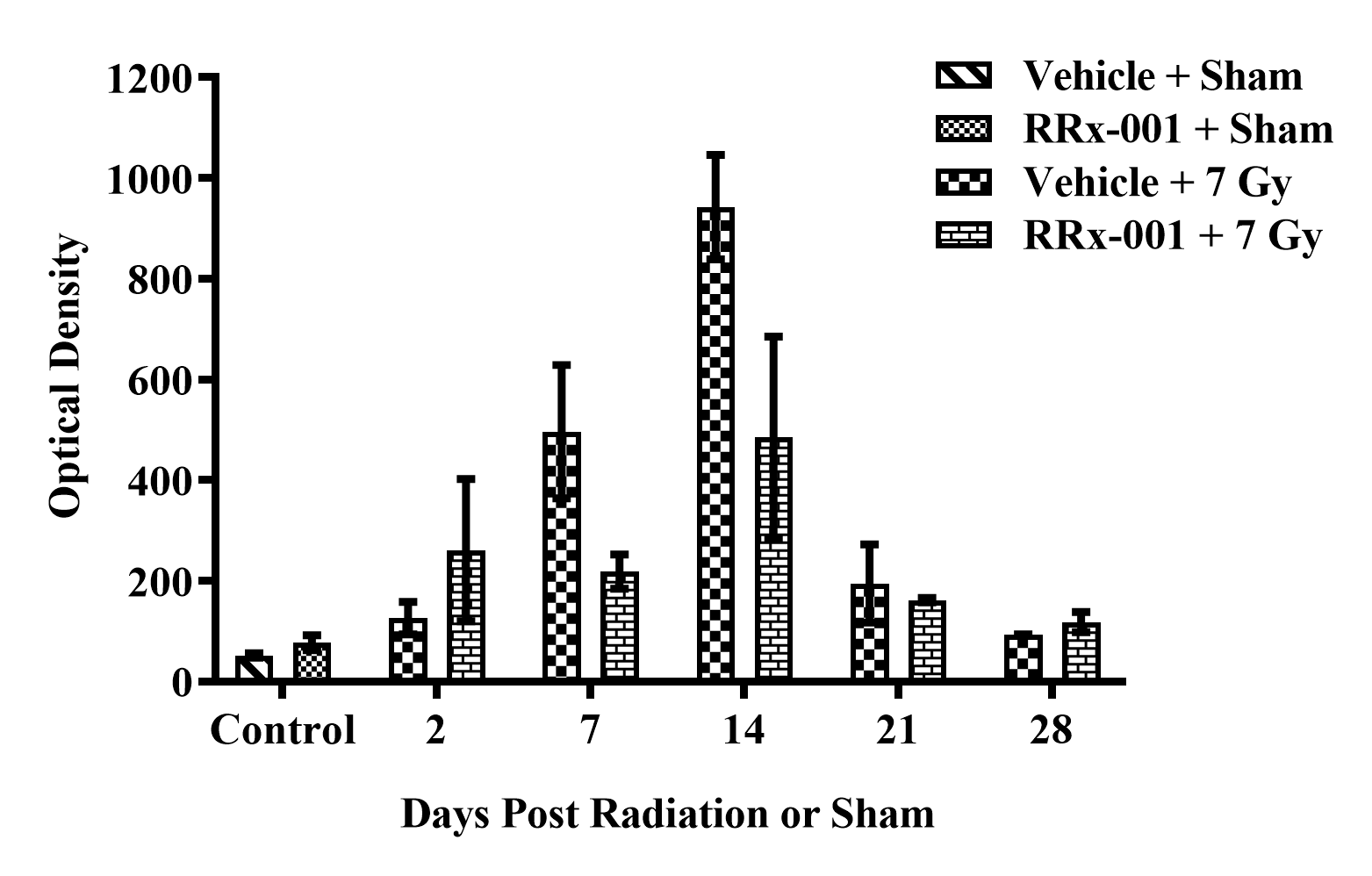

Supplement: Supplementary file 1 [file image3.tif]

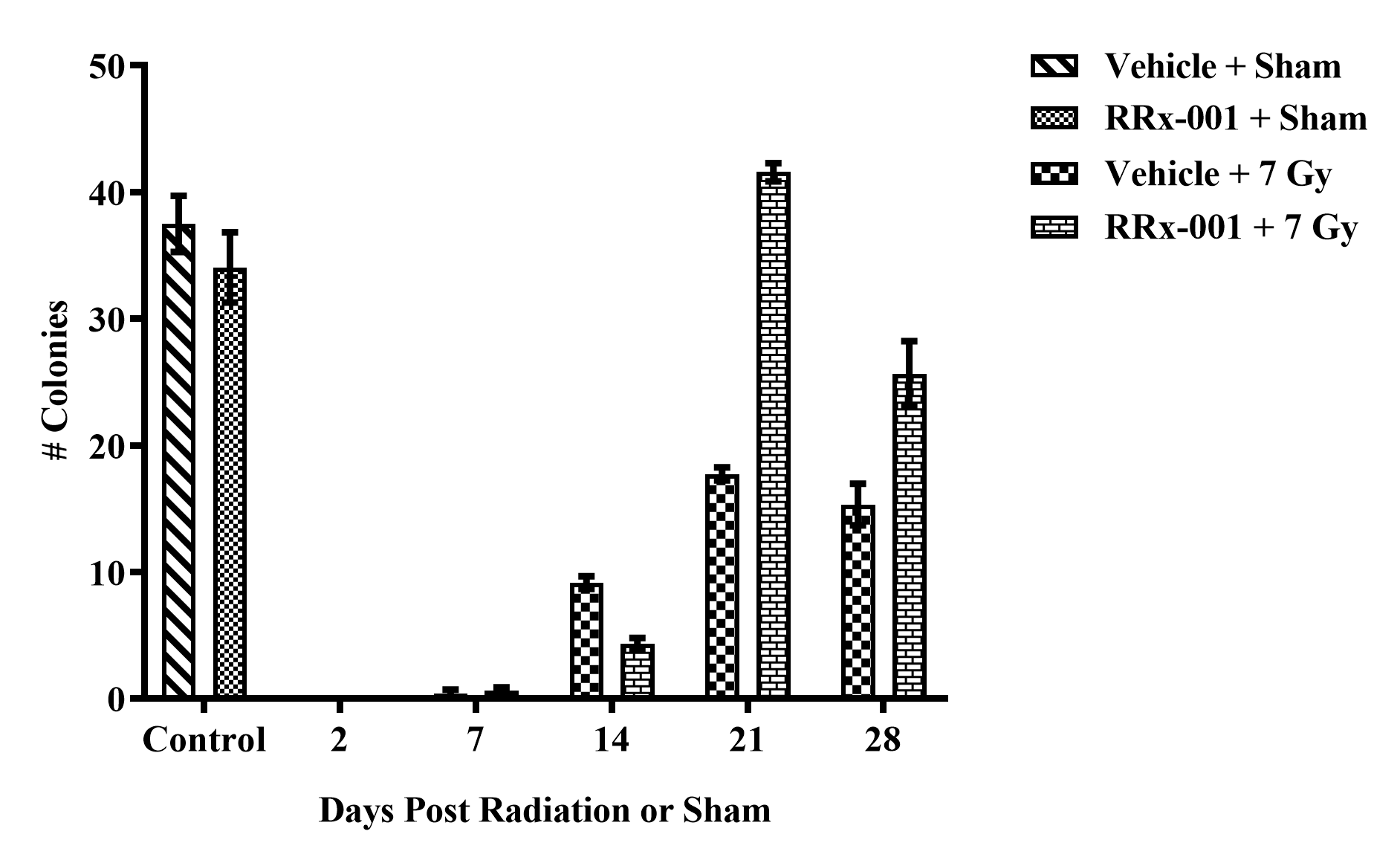

Supplement: Supplementary file 2 [file image2.tif]

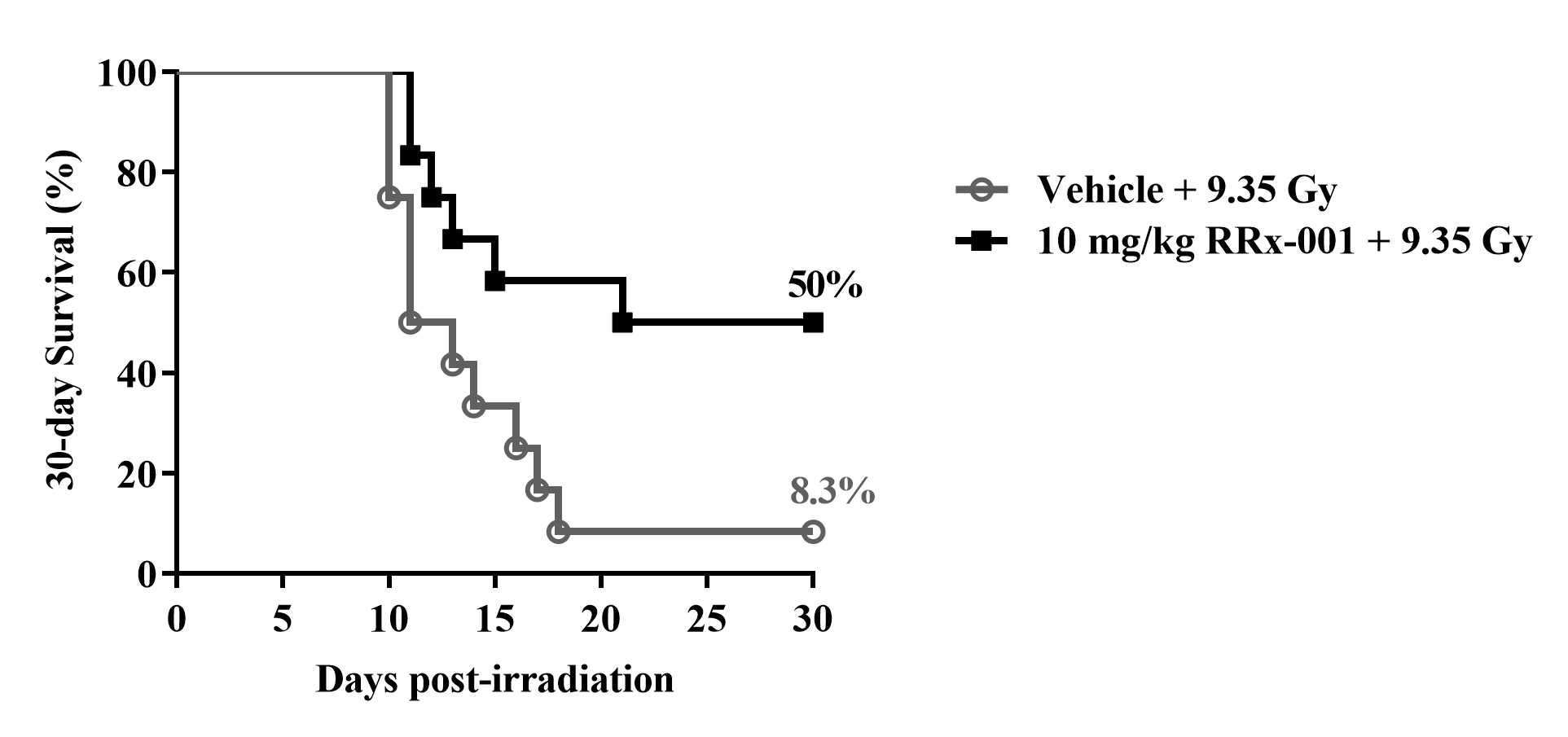

Supplement: Supplementary file 3 [file image1.tif]
