# Supplementary material for: RRx-001 Radioprotection: Enhancement of Survival and Hematopoietic Recovery in Gamma-Irradiated Mice
Source: Front Pharmacol. 2021 Apr 22;12:676396. doi: 10.3389/fphar.2021.676396 (PMC8100686; doi:10.3389/fphar.2021.676396)
Supplement: Supplementary file 4 [file datasheet1.pdf]

Supplementary Material

Supplementary Figure 1. Kaplan-Meier 30-day survival curves illustrating the prolonged survival of CD2F1 mice prophylactically treated by IV injection with 10 mg/kg RRx-001 compared to the vehicle control, 24 hours prior to 9.35 Gy whole body irradiation; log-rank $\chi^2_{(1)}$ = 5.62, *p*=.018. N = 12 mice/group (24 mice total).

Supplementary Figure 2. An increase in number of colonies, thus an increase in hematopoietic progenitor clonogenicity, of mouse bone marrow cells was observed in the sublethally irradiated groups prophylactically treated with 10 mg/kg RRx-001 compared to the vehicle control on and after day 21. The bone marrow was pooled for each group (n = 3/group/timepoint) and plated in triplicate. All colonies (one colony = 50+ cells) were counted 12 days after incubation. The sham-irradiated RRx-001- and vehicle-pretreated groups show all time points combined and graphed as mean ± SEM; n = 12 mice for the vehicle group (sample for day 21 was lost) and n = 15 mice for the RRx-001 group. Irradiated RRx-001- and vehicle-pretreated groups are shown as the mean ± SEM of n = 3 mice/group/time point. Bone marrow collected from 57 mice was used in the experiment.

Supplementary Figure 3. G-CSF levels in the serum of sublethally irradiated mice prophylactically treated with 10 mg/kg RRx-001 did not increase to the levels seen in the vehicle control group on day's 7 and 14. Sham-irradiated RRx-001- and vehicle-pretreated groups show all time points combined and graphed as mean ± SEM. Irradiated RRx-001- and vehicle-pretreated groups are shown as the mean ± SEM of n = 2 - 3 mice/group/time point. Serum from 50 mice was used for the experiment.
